# The effect of the fenugreek hydrolyzed protein on lipid profile in patients with mild-to-moderate hypercholesterolemia: A confirmatory triple-blind randomized-controlled clinical trial

**DOI:** 10.1016/j.phyplu.2024.100691

**Published:** 2025-02

**Authors:** Mahdi Badiee Gavarti, Ali Askari, Hamidreza Roohafza, Mozhde Askari, Zahra Teimouri Jervekani, Shima Kaveh, Mohammad Kermanialghoraishi, Alireza Sadeghimahoonak, Masoumeh Sadeghi

**Affiliations:** a Student Research Committee, School of Medicine, Isfahan Cardiovascular Research Center, Cardiovascular Research Institute, Isfahan University of Medical Sciences, Isfahan, Iran; b Cardiac Rehabilitation Research Center, Cardiovascular Research Institute, Isfahan University of Medical Sciences, Isfahan, Iran; c Isfahan Cardiovascular Research Center, Cardiovascular Research Institute, Isfahan University of Medical Sciences, Isfahan, Iran; d Faculty of Food Science & Technology, Gorgan University of Agricultural Sciences and Natural Resources, Gorgan, Iran; e Interventional Cardiology Research Center, Cardiovascular Research Institute, Isfahan University of Medical Sciences, Isfahan, Iran; f Department of Food Science and Technology, Gorgan University of Agricultural Sciences, Gorgan, Iran

**Keywords:** Fenugreek, Hypercholesterolemia, Blood lipids

## Abstract

**Background:**

The risk of coronary artery disease in people with high serum cholesterol is more than twice as high as in those with moderate serum cholesterol. Natural medicines, especially herbs, have been the focus of attention for many years because of the desirable and minimal side effects for controlling blood lipids.

**Purpose:**

The present study aims to investigate the effect of fenugreek hydrolyzed protein (FHP) on lipid profile in patients with mild-to-moderate hypercholesterolemia.

**Methods:**

This study is a confirmatory, triple-blind, two-group parallel, randomized controlled trial, phase 3 conducted on patients aged 18–65 years with mild-to-moderate hypercholesterolemia and low risk for cardiovascular disease. Patients were recruited from a private clinic from May 2021 to June 2021. Sixty patients were randomized with a 1:1 allocation ratio into the FHP group (N = 30) and the placebo group (N = 30). The intervention group was administrated 40 mg/day of FHP for 8 weeks. Patients were examined at baseline and 8 weeks after randomization. The primary outcome was the patient's lipid profile including total cholesterol (TC), high-density lipoprotein (HDL-c), low-density lipoprotein (LDL-c), triglyceride (TG), and non-HDL-c. All participants, caregivers, outcome assessors, and analyzers were blind to the type of intervention.

**Results:**

In the intervention group, lipid profile improved through a significant reduction in TC (P < 0.001), LDL-c (P = 0.043), and non-HDL-c (P < 0.001), but no significant changes were observed in the TG level. The mean difference of these variables was -10.07 mg/dl [95 %CI:30.84; 10.70], -8.93 mg/dl [95 %CI:27.07; 9.21], and -10.37 mg/dl [95 %CI:32.26; 11.52] for TC, LDL-c, and non-HDL-c, respectively. FHP successfully decreased LDL-c level by 7 %. There was no significant change in any component of the lipid profile in the comparison group. FHP was well tolerated with only 1 patient experiencing gastrointestinal adverse manifestations.

**Conclusion:**

These findings suggest that FHP administration can improve the lipid profile of patients with mild-to-moderate hypercholesterolemia. Considering the low adverse effects of FHP and patients' high tolerance, it can be considered in the management of these patients, who fall into the low-risk cardiovascular disease category, adjuvant to the main therapies.

**Registration code:**

IRCT20210125050 142N1

## Highlights

• Fenugreek Hydrolyzed Protein (FHP) can statistically significantly improve lipid profile.
• FHP can reduce serum LDL-c levels by 7 % in patients with mild hypercholesterolemia.
• The efficacy of FHP in controlling blood lipids is small in magnitude.
• LDL-c serum levels were successfully reduced to below 130 mg/dl by FHP administration.
• FHP has low adverse effects and is well tolerated.

## Graphical abstract

**Figure:**
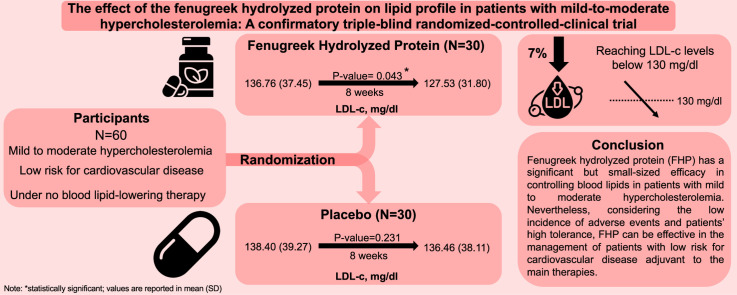

**Abbreviations**
ACS: Acute Coronary Syndrome
BMI: Body Mass Index
CVD: Cardiovascular Disease
CAD: Coronary Artery Disease
DBP: Diastolic Blood Pressure
FHP: Fenugreek Hydrolyzed Protein
HDL-c: High-Density Lipoprotein cholesterol
LDL-c: Low-Density Lipoprotein cholesterol
SBP: Systolic Blood Pressure
TC: Total Cholesterol
TG: Triglyceride

## 1 Introduction

Atherosclerotic cardiovascular disease is considered to be the leading cause of mortality worldwide (Barquera et al., 2015). The risk of coronary artery disease (CAD) in people with high serum cholesterol is more than twice as high as those with moderate serum cholesterol. Evidence has shown that lowering blood lipids through diet or drug interventions slows the progression of atherosclerosis and, ultimately, the incidence of CAD (Hadaegh et al., 2009). According to the results of the UK Simon Broome Register, the coronary heart disease mortality rate among treated hypercholesterolemia patients has decreased by more than one-third (Sharifi et al., 2019). Among serum lipids, low-density lipoprotein cholesterol (LDL-c) is also most associated with atherosclerosis (Azizi et al., 2004). Except when triglyceride (TG) is elevated, most plasma cholesterol is transported by LDL. Therefore, lowering LDL-c in high-risk patients is clinically beneficial (Tannock, 2008).

Considering all the cardiovascular risk factors, the studies’ findings show that the control of blood lipids can reduce the incidence of ischemia. In this regard, statins are used as drugs for treating dyslipidemia, especially hypercholesterolemia. Apart from the beneficial effects of statins, these drugs also have many side effects, including myopathy and rhabdomyolysis, liver complications, myasthenia gravis, nausea, dizziness, and gastrointestinal problems (Imperato, 2002). Since blood lipids lowering and controlling drugs have undesirable side effects, researchers are looking for a way out. Therefore, natural medicines, especially herbs, have been the focus of attention for many years. Plants contain bioactive compounds and phytochemicals including phenolic, flavonoid, and anthocyanin compounds. Epidemiologic studies have shown that the consumption of foods and beverages containing bioactive compounds may reduce the risk of cardiovascular disease (Kaur et al., 2018). In recent years, hydrolyzed proteins with antioxidants and health-promoting properties have been produced from many animal and plant sources such as milk, soybeans, canola, etc. However, plant sources have received more attention due to their lower cost and lower allergen city (Denke and Grundy, 1996).

Among the plant sources suitable for producing bioactive peptides, Fenugreek seed (*Trigonella foenum-graecum*), from the butterfly family and the legume family has many beneficial effects including hypoglycemic effect in diabetes mellitus, blood lipid-lowering, blood pressure-lowering, analgesic, anti-bloating, anti-cancer, male libido enhancement, breastfeeding, anti-worm, uterine tonic, and facilitating labor ((US) et al., 1993). Fenugreek dietary fibers bind to glucose and cholesterol after eating and regulate the production of cholesterol in the liver; furthermore, it stimulates the formation of bile in the liver and the conversion of cholesterol into bile acid. The viscosity of the digestive system decreases cholesterol and bile acid absorption, and the production of short-chain fatty acids by fiber fermentation, which seems to prevent hepatic cholesterol synthesis; These fibers in the water emulsify and stabilize foods due to the presence of galactomannan compounds (Kirklin et al., 2002).

Several previous animal studies have shown the beneficial effect of fenugreek on lipid profile. A study of 24 rats fed fenugreek seed or leaf for eight weeks showed a statistically significant effect on lipid profile improvement, antioxidant activity, and performance improvement (Wani and Kumar 2018). Another study of 20 albino rabbits induced cholesterol for eight weeks, followed by a fenugreek seeds injectable emulsion powder administration, showed that their lipid profile and body weight were statistically significantly decreased, and serum high-density lipoprotein cholesterol (HDL-c) levels were also increased (Mathew and Abraham, 2006).

Fenugreek's effect was also previously investigated in human subjects. In a study of 61 patients with the American Heart Association's (AHA) step 1 diet, the intervention group received 60 gs of fenugreek seed per day and the comparison group received 1 gram of cellulose for 12 weeks which resulted in a statistically significant decrease in lipid profile and fasting plasma glucose (FPG), and postprandial blood glucose. There was a decrease in serum HDL-c levels (Dohadwala and Vita, 2009). In a previous study in Iran on 56 patients with borderline hyperlipidemia, the intervention group received 8 gs of fenugreek egg sachet for 8 weeks. A significant decrease in lipid profile was seen, but a decrease in body mass index (BMI) and an increase in serum HDL-c levels were not significant (Sun et al., 2004).

The cost and side effects reported from different studies advised herbal ingredients will have both less therapeutic costs and far fewer side effects and surely better medication use (Mathew and Abraham, 2006). Accordingly, assessing the fenugreek efficacy and safety as an herbal intervention seems beneficial. To the best of our knowledge, a hydrolyzed protein of the fenugreek seeds has never been administrated to dyslipidemic patients and our knowledge of its efficacy and safety is scarce. Therefore, the present study aims to investigate the effect of fenugreek hydrolyzed protein (FHP) on the lipid profile of hypercholesterolemia patients and its potential adverse effects.

## 2 Methods

This study is a confirmatory, triple-blind, two-group parallel, randomized-controlled clinical trial conducted on patients with mild to moderate hypercholesterolemia. All participants were recruited from a private clinic under the cover of Isfahan University of Medical Sciences at Isfahan. Informed consent was obtained from each subject and they were debriefed during the process for using their medical data anonymously for research goals. Laboratory tests and drugs were entirely free for patients. The manuscript is written in adherence to the Consolidated Standards of Reporting Trials (CONSORT) 2010 reporting guideline and its extensions, CONSORT for abstracts, and CONSORT herbals (Gagnier et al., 2006; Hopewell et al., 2008). The proposal of the present trial was approved in 2020 by the National Institute for Medical Research Development (NIMAD) and the protocol was prospectively registered in 2020 on the Iranian Registry of Clinical Trials (IRCT) (IRCT20210125050 142N1).

### 2.1 Participants

Patients aged 18–65 years with low to moderate risk for cardiovascular events. According to the European Society of Cardiology, patients with LDL-c levels lower than 116 mg/dl are at low risk. Accordingly, LDL-c levels of >116 mg / dL and <180 mg / dL were eligible. Patients with high and very high risk for cardiovascular events, those who need lipid-lowering drugs with the aim of secondary prevention (documented cardiovascular diseases), LDL-c levels equal to and greater than 180 mg/dL requiring drug treatment, drinking alcohol, use of effective supplements on blood lipids such as fish oil, use of immunosuppressive drugs, blood lipid-lowering drugs (statins, fibrates, niacin (Niacin), etc.), patients with hypothyroidism, nephrotic syndrome or renal dysfunction or liver dysfunction, patients with uncontrolled hypertension (systolic blood pressure (SBP) greater than 160 mmHg or diastolic blood pressure (DBP) of 100 mmHg)), history of dizziness and convulsions, pregnancy, or lactation were not included in this study. Also, Patients with non-compliance to prescribed medications for >1 week were excluded from the study (Kelishadi et al., 2008).

### 2.2 Randomization & intervention

By using the patient's ID in a random number table and according to the inclusion and exclusion criteria samples were selected from the target population. Randomization was executed each time for each patient recruited using SAS 9.2 statistical software PROC PLAN with a non-blocking approach. The sequence generation was carried out by a third person out of the study who was asked about the patient's group allocation by the care provider (author MS) through the telephone. Accordingly, no allocation concealment was needed. Subjects were randomized into two groups of 30 patients with a 1:1 allocation ratio. In the comparison group, 30 subjects received 40 mg of the placebo daily, and in the intervention group, 30 patients received 40 mg of FHP daily for 8 weeks. Both prescriptions were synthesized from Gorgan University. The diet and lifestyle recommended to each group will be the same.

### 2.3 Blinding

The outcome assessor, care provider, data analyzer, and patients were blinded to the type of drugs. All patients received the prescriptions from a physician as the care provider (author MS) face to face. The appearance of the packages and the tablets of FHP and the placebo were the same.

### 2.4 Primary outcome

The blood samples of patients were obtained at the baseline and after 8 weeks from the randomization. Serum samples from all subjects were collected, and blood lipid levels (total cholesterol (TC), HDL-c, LDL-c, TG, and non-HDL-c) were measured in the central laboratory.

### 2.5 Secondary outcomes

On two occasions, individuals are examined for height, weight, and blood pressure. The height and weight of people without shoes were measured by an expert using scales and tape gauges, and the BMI was calculated from the weight (kg) divided by the square of the height (m). Also, the FPG level was measured through blood samples in the central laboratory.

### 2.6 Post-hoc outcomes

To address the safety of this intervention, we evaluated potential adverse events in patients following FHP administration in addition to outcomes pre-specified in the protocol.

### 2.7 Preparation of fenugreek hydrolyzed protein and placebo capsules

The herbal source was the hydrolyzed protein of Fenugreek (*Trigonella foenum-graecum*) seed. Dried Fenugreek seed was powdered and defatted using hexane, and then protein was extracted by isoelectric precipitation. Alkalase and trypsin enzymes were used to prepare protein hydrolysate from fenugreek protein concentrate under optimum conditions of activity of each enzyme. Then, the characteristic of protein hydrolysate was evaluated by DPPH free radical scavenging activity, ferric reducing power, Fe chelating activity, and amino acid composition. Finally, the best sample was selected.

Fenugreek seed protein hydrolysate or unhydrolyzed protein was mixed with starch, and the resulting mixture was filled in the empty capsules. The placebo was made from starch and was similar to other capsules in color and quantity. The final concentration of protein or protein hydrolysate used was 40 mg/capsule.

### 2.8 Priori sample size calculation

According to the primary outcome of this study (blood serum lipids), which are of a small continuous type, the t-student formula is used to measure the sample size.

α error of 5 %, β error of 20 %, and minimum clinical important difference (MCID) of 10 mg/dl were considered. According to the literature, the standard deviation (SD) for LDL-c levels was 15.47 mg/dl (Kelishadi et al., 2008). Considering the potential attrition of 20 %, each intervention and comparison group must have 30 participants.

### 2.9 Statistical analysis

Data are presented as mean (SD) for continuous variables and counts and percentages for categorical variables. Student *t*-test was used for continuous and Chi-square (Fisher exact) test for categorical data. Paired t-test was used to compare before and after groups. Mean difference and Morris d_ppc2_ (as a standardized mean difference) were calculated with STATA version 14 (StataCorp. (2015)) and Campbell web-based effect size calculator, respectively (Morris, 2008; Wilson, D. B., n.d.). The interpretational zones used for d_ppc2_ are as follows: 0 to 0.19 “trivial”, 0.2 to 0.49 “small”, 0.5 to 0.79 “medium”, 0.8 to 0.99 “large”, and > 1 “very large” size of efficacy (Kraemer et al., 2003). All other statistical analyses were performed using SPSS 20.0 (IBM. Corp., Armonk, NY, USA). *P* < 0.05 was considered statistically significant.

### 2.10 Ethics

Written informed consent was obtained from all participants after the project was fully explained. All paid services were entirely free for patients. The study was approved by the National Ethics Committee on Iranian Biological Research.

## 3 Results

**Fig. 1:**
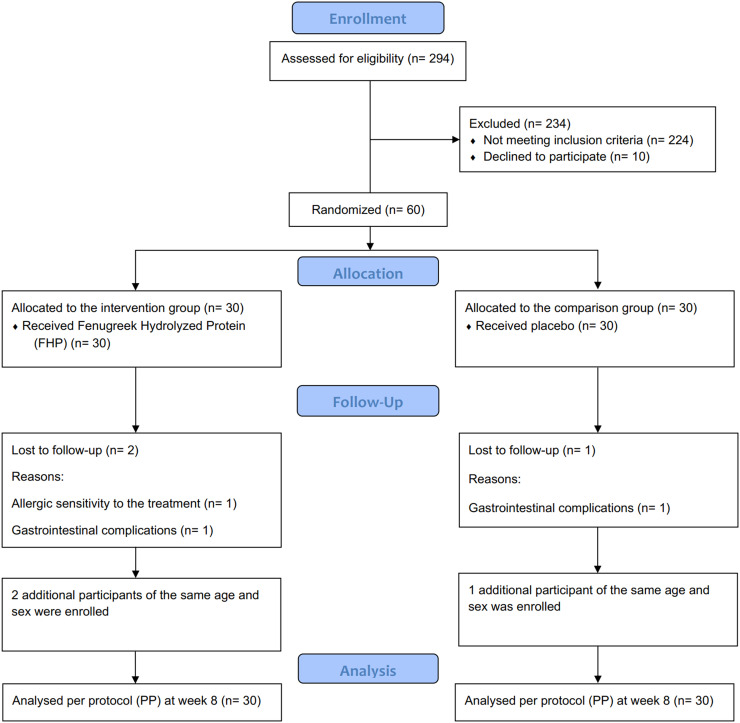
Trial flow diagram.

A total number of 294 patients were evaluated for the possible inclusion. One hundred and eighteen patients had a history of dyslipidemia and their lipid profile were further investigated by blood tests. Seventy patients were eligible but only 60 patients were enrolled due to non-participation. Eventually, a total number of 60 participants were randomized into the intervention (N = 30) and comparison (N = 30) group between May 2021 to June 2021 recruited from a private clinic under cover of Isfahan University of Medical Sciences and were followed up for 8 weeks. The CONSORT flow diagram of the trial is presented in Fig. 1.

### 3.1 Baseline characteristics

The mean age of patients was 45.62 ± 6.72 and 49.26 ± 8.72 years in the FHP group and the placebo group, respectively. Among the patients, 70 % were female, of which 44 % were in the placebo group and 53 % were in the intervention group. The baseline anthropometric and clinical characteristics of the patients in the two groups are presented in Table 1.

**Table 1:** Baseline anthropometric and clinical characteristics.

	All Participants (N = 60)	FHP group (N = 30)	Placebo group (N = 30)
sex (woman %)	70	53	44
Age, years	47.39 (7.84)	45.62 (6.72)	49.26 (8.72)
Waist circumference, cm	86.77 (8.56)	88.13 (9.44)	85.40 (4.05)
Weight, Kg	74.53 (10.64)	75.33 (13.55)	73.73 (7.04)
Hip circumference, cm	100.27 (5.16)	100.53 (6.06)	100.00 (4.27)
Height, cm	164.63 (9.70)	168.0 (10.17)	160.73 (7.48)
BMI, kg/m^2^	27.58 (3.40)	26.42 (3.60)	28.60 (3.01)
WHR	0.86 (0.06)	0.87 (0.05)	0.85 (0.06)
SBP, mm Hg	113.33 (6.21)	114.00 (6.32)	112.66 (6.22)
DBP, mm Hg	75.50 (5.14)	75.66 (5.6)	75.33 (4.80)
TC, mg/dL	228.90 (42.38)	225.13 (44.46)	232.66 (41.37)
TG, mg/dL	163.97 (76.97)	174.40 (89.69)	153.53 (63.18)
LDL-C, mg/dL	141.10 (42.26)	136.60 (38.01)	145.60 (47.02)
HDL-C, mg/dL	40.17 (8.99)	40.80 (8.63)	39.53 (9.58)
FPG, mg/dL	92.00 (13.75)	95.80 (15.95)	88.20 (10.29)
Non-HDL-C, mg/dL	188.73 (43.62)	184.3 (44.22)	193.1 (44.09)

Note: Continuous variables are reported in mean (SD); BMI= body mass index, DBP =diastolic blood pressure, FPG =fasting plasma glucose, HDL-C =high-density lipoprotein cholesterol, LDL-C =low-density lipoprotein cholesterol, non-HDL-C =non-high-density lipoprotein cholesterol, SBP =systolic blood pressure, WHR =waist-to-hip ratio, TC =total cholesterol, TG =triglycerides, FHP= fenugreek hydrolyze protein.

### 3.2 Confirmatory results (lipid profile)

At week 8, there was a significant reduction in TC (P < 0.001), LDL-c (P = 0.043), and non-HDL-c (P < 0.001) levels in the intervention group. The most declining changes occurred in TC (20 mg/dl) and non-HDL-c (16.97 mg/dl). FHP reduced LDL-c level by 7 %. The HDL-c level was enhanced non-significantly from 40.96 mg/dl to 41.93 mg/dl in the intervention group. No significant reduction in any blood lipid was observed in the comparison group (Table 2). There was no significant change in TG level in both groups. FHP has reduced TC, LDL-c, and non-HDL-c serum levels by 10.07 mg/dl, 8.93 mg/dl, and 10.37 mg/dl respectively compared to the placebo group. d_ppc2_ measure, as a difference-in-difference standardized effect size, shows a trivial and inconsiderable effect of FHP on TG and HDL-c serum levels. However, FHP has a small-sized decreasing efficacy on TC, LDL-c, and non-HDL-c levels; mean differences and d_ppc2_ of previously mentioned outcomes are also illustrated in Table 2.

**Table 2:** Effect of fenugreek hydrolyze protein administration on lipid profile after 8 weeks.

	Before FHP (N = 30)	After FHP (N = 30)	Intra-group P-Value	Mean change percentage^†^	Before Placebo (N = 30)	After Placebo (N = 30)	Intra-group p-Value	Inter-group p-value	Mean difference	95 %CI	d_ppc2_	95 %CI
TC, mg/dL	231.86 (44.60)	211.86 (36.13)	< 0.001*	−4.75 %	225.60 (45.69)	221.93 (43.86)	0.136	0.168	−10.07	−30.84; 10.70	−0.38	−0.90; 0.15
TG, mg/dL	168.83 (80.02)	158.00 (92.34)	0.110	−6.42 %	169.53 (86.18)	170.93 (93.33)	0.735	0.296	−12.93	−60.91; 35.05	−0.14	−0.66; 0.38
LDL-c, mg/dL	136.76 (37.45)	127.53 (31.80)	0.043*	−7 %	138.40 (39.27)	136.46 (38.11)	0.231	0.164	−8.93	−27.07; 9.21	−0.2	−0.72; 0.32
HDL-c, mg/dL	40.96 (9.54)	41.93 (9.32)	0.286	2.37 %	41.46 (8.71)	41.50 (9.10)	0.946	0.429	0.43	−4.33; 5.19	0.10	−0.42; 0.62
Non-HDL-c, mg/dL	187.03 (43.18)	170.06 (38.05)	< 0.001*	−6.1 %	184.13 (48.43)	180.43 (46.26)	0.149	0.174	−10.37	−32.26; 11.52	- 0.30	−0.82; 0.22

Note: All variables are reported in mean (SD); * statistically significant † percentage of mean change in serum levels compared to the baseline; HDL-*c* =high-density lipoprotein cholesterol, LDL-*c*= low-density lipoprotein cholesterol, non-HDL-*c*= non-high-density lipoprotein cholesterol, TC =total cholesterol, TG= triglycerides, FHP= fenugreek hydrolyze protein.

### 3.3 Exploratory results

Placebo use showed a slight change in weight (P = 0.053, borderline), and BMI (P = 0.051, borderline) in the comparison group (Table 3). In the FHP group, a significant change in Weight (P < 0.001), BMI (P < 0.001), SBP (P = 0.025), and DBP (P < 0.001) was seen. No significant change in FPG was observed in both groups. d_ppc2_ measure shows a trivial and inconsiderable effect of FHP on Weight, BMI, waist circumference, hip circumference, and FPG. However, FHP can reduce SBP and DBP with a small-sized efficacy.

**Table 3:** Effect of fenugreek hydrolyze protein administration on secondary outcomes after 8 weeks.

	Before FHP (N = 30)	After FHP (N = 30)	Intra-group p-Value	Before Placebo (N = 30)	After Placebo (N = 30)	Intra-group p-Value	Inter-group p-value	Mean difference	95 %CI	d_ppc2_	95 %CI
Waist circumference, cm	86.76 (8.55)	86.60 (8.53)	0.096	86.76 (8.55)	86.70 (8.51)	0.161	0.482	−0.1	−4.50; 4.30	−0.01	−0.53; 0.50
Hip circumference, cm	100.23 (5.15)	100.03 (5.20)	0.310	100.20 (5.14)	100.20 (5.14)	0.999	0.450	−0.17	−2.84; 2.50	−0.04	−0.55; 0.48
Weight, kg	74.56 (10.61)	73.83 (10.87)	<0.001*	74.36 (10.54)	74.16 (10.58)	0.053	0.453	−0.33	−5.87; 5.21	−0.05	−0.57; 0.47
BMI, kg/m2	27.53 (3.44)	27.24 (3.41)	<0.001*	27.46 (3.46)	27.39 (3.49)	0.051	0.433	−0.015	−1.93; 1.63	−0.06	−0.58; 0.45
SBP, mm Hg	112.58 (5.7)	110.68 (5.93)	0.025*	113.33 (6.06)	112.83 (6.39)	0.639	0.09	−2.15	−5.33; 1.03	−0.23	−0.75; 0.29
DBP, mm Hg	75.66 (5.04)	74.16 (4.7)	<0.001*	75.50 (4.97)	75.33 (5.07)	0.326	0.179	−1.17	−3.70; 1.36	−0.27	−0.79; 0.25
FPG, mg/dL	94.53 (14.09)	94.50 (14.19)	0.981	92.03 (14.38)	91.73 (12.92)	0.631	0.784	2.77	−4.24; 9.78	0.02	−0.50; 0.54

Note: All variables are reported in mean (SD); * statistically significant; FHP=fenugreek hydrolyzed protein, BMI =body mass index, DBP= diastolic blood pressure, FPG =fasting plasma glucose, SBP= systolic blood pressure.

### 3.4 Post-hoc results

#### 3.4.1 Adverse effects

FHP was well tolerated with only one patient experiencing gastrointestinal complications and one with allergic hypersensitivity reactions.

## 4 Discussion

In the present study, we revealed the hypocholesterolemic effect of FHP by administering 40 mg/day of FHP to mild to moderate hypercholesterolemia patients significantly reducing TC, LDL-c, and non-HDL-c of lipid profile and SBP and DBP with a small-sized efficacy. This significant effect is shown by paired t-test within the FHP group but the student t-test between FHP and placebo group shows no significant results. This may originate from the placebo effect attenuating the FHP efficacy resulting in non-significant results. However, considering the before-after essence of lipid-lowering trials deriving from the clinical management of an individual patient's lipid profile, we assume that the paired t-test can be more reliable in the interpretation of our results. We also included effect size measures in our judgment which confirm the significant results.

There are several previously published animal studies investigating the effect of fenugreek on lipid profile and the findings are very heterogeneous. Two studies on hamsters and rats with high-cholesterol diet and metabolic syndrome, respectively, showed a significant decrease in TC and LDL-c levels (Kassaee et al., 2021; Mohammad-Sadeghipour et al., 2020). In contrast, another study on rats with both a high-fat high-sucrose diet and metabolic disorder showed no significant difference in TC (Muraki et al., 2011). Accordingly, the judgment of fenugreek efficacy in controlling lipid profile is challenging and a meta-analysis of animal studies seems necessary to reach more conclusive results.

Even though the lipid profile-controlling properties of fenugreek have been comprehensively evaluated in animal studies, a precise investigation in human subjects is still lacking. In a study on 30 CAD patients administrated 5 g of fenugreek daily for 3 months, Fenugreek treatment significantly decreased TC and TG after 3 months with no significant effect after 1.5 months (Bordia et al., 1997). Another study was carried out with a daily dose of 12.5 g and 18.0 g of germinated fenugreek seed administrated to two groups of vegetarian patients with hypocholesterolemia for one month. Results show a significant reduction in the levels of TC and LDL-c in both groups. In concordance with our results, no significant reduction in TG serum levels was noticeable (Sowmya and Rajyalakshmi, 1999). Therefore, these results may imply that a longer period of FHP administration may be necessary for its significant effect on TG but its effect on TC and LDL-c may be seen sooner. Our findings also reveal a non-significant decrease in TG levels but a significant decrease in TC levels after 2 months in patients with hypercholesterolemia. Accordingly, further research with a longer follow-up period and repeated measurements is required to establish whether longer fenugreek treatment has better efficiency in controlling TG and cholesterol levels in both hypercholesterolemia and CAD patients and evaluate its latent efficacy. Additionally, a triple-blind randomized controlled clinical trial was conducted on type 2 diabetic patients evaluating the efficacy of fenugreek seed in controlling serum metabolic factors. After administration of 10 g/d fenugreek whole seed for 8 weeks, patients showed a significant reduction in TC and TG but trivial differences in LDL-C and HDL-C (Rafraf et al., 2014). In line with our findings, TC is decreased with almost the same magnitude by fenugreek in both diabetic and hypercholesterolemic patients. However, TG had the most prominent reduction in diabetic patients in contrast to our trial with trivial changes in hypercholesterolemia. On the other hand, LDL-C was more pronouncedly decreased in our population compared to diabetes mellitus. Taken together, it seems that fenugreeks’ efficacy is reliable to patients’ baseline metabolic condition showing higher efficacy in higher baseline levels of each lipid component with TG in diabetic patients having metabolic syndrome and LDL-C in hypercholesterolemic patients. Future clinical trials on patients with different medical conditions are recommended to expand our knowledge of fenugreek's potential efficacy in each group.

There are several definitions for minimal clinically important difference (MCID) of LDL-c and there is no agreement on which is the ultimate one. we evaluated the clinical significance of our intervention noting that this trial was on patients with mild to moderate hypercholesterolemia. Accordingly, considering an MCID definition of reaching LDL-c levels goal of lower than 130 mg/dl in patients with low-risk for cardiovascular disease (CVD), FHP could successfully achieve it and is considered to be clinically significant for this population (Besseling et al., 2013). However, our findings imply that FHP has a trivial and inconsiderable effect on TG, HDL-c, weight, BMI, and FPG. Moreover, further studies evaluating the clinical significance of FHP in patients with higher baseline levels of LDL-c are necessary.

According to AHA 2018 guidelines on the management of blood cholesterol, our study sample falls into the low-risk CVD category. The guideline's recommendation for this category is to emphasize lifestyle modifications in order to reduce risk factors (Grundy et al., 2019). Considering the low incidence of adverse events (n = 2 (6.67 %)) after administration of FHP and the patient's high tolerance, it may be beneficial in the primary prevention of these patients as an adjuvant to AHA's recommendation.

This study's results show that LDL-c level reduction accounts for 89 % of TC level depletion by FHP administration. Accordingly, FHP is mostly effective in lowering LDL-c among all lipid profile components. Considering the pivotal role of LDL-c in the pathogenesis of atherosclerotic plaque formation, coronary artery stenosis, and consequently acute coronary syndrome (ACS) occurrence, FHP might be favorably effective in ACS primary prevention (Brown and Goldstein, 1986; Gao and Liu, 2017).

To address the limitations of this study, we should point out that the 95 % confidence intervals of our effect sizes are pretty wide and these results are inconclusive. Therefore, clinical trials with greater sample size or meta-analysis may attain satisfying power in order to achieve conclusive results with a more definitive demonstration of internal validity. Spite the exclusive eligibility criteria of the trial, future trials with adequate power may benefit from adjusted analysis for potential confounders. Additionally, possible differences in response to treatment of hypercholesterolemia patients should be evaluated in subgroup analysis in future trials with larger sample sizes. Further equivalence trials are needed to confirm and approve the lipid profile controlling property of fenugreek on human subjects and compare its effect with gold-standard therapies including statins, especially in the low-risk population (Grundy et al., 2019).

## 5 Conclusion

In summary, this trial's findings imply that FHP has a statistically significant decreasing effect on serum TC, LDL-c, and non-HDL-c of patients with mild-to-moderate hypercholesterolemia. Despite that FHP efficacy is low in magnitude, considering the low incidence of adverse events, it may be beneficial in the primary prevention of these patients, with a low risk for CVD, as an adjuvant to the main therapies.

## Funding

Research reported in this publication was supported by the Elite Researcher Grant Committee under award number 988,159 from the National Institute for Medical Research Development (NIMAD), Tehran, Iran.

## CRediT authorship contribution statement

**Mahdi Badiee Gavarti:** Writing – review & editing, Writing – original draft, Validation, Funding acquisition, Formal analysis, Data curation. **Ali Askari:** Writing – review & editing, Writing – original draft, Visualization, Validation, Formal analysis, Data curation. **Hamidreza Roohafza:** Writing – review & editing, Visualization, Supervision, Methodology, Formal analysis. **Mozhde Askari:** Writing – review & editing, Methodology. **Zahra Teimouri Jervekani:** Writing – review & editing, Methodology. **Shima Kaveh:** Writing – review & editing, Validation, Methodology. **Mohammad Kermanialghoraishi:** Writing – review & editing, Methodology. **Alireza Sadeghimahoonak:** Writing – review & editing, Validation, Resources, Project administration, Methodology, Investigation, Conceptualization. **Masoumeh Sadeghi:** Writing – review & editing, Visualization, Validation, Supervision, Resources, Project administration, Methodology, Investigation, Funding acquisition, Conceptualization.

## Declaration of competing interest

The authors declare that they have no known competing financial interests or personal relationships that could have appeared to influence the work reported in this paper.
